# The choroid-sclera interface: An ultrastructural study

**DOI:** 10.1016/j.heliyon.2022.e09408

**Published:** 2022-05-10

**Authors:** C. Platzl, A. Kaser-Eichberger, A. Benavente-Perez, F. Schroedl

**Affiliations:** a Center for Anatomy and Cell Biology, Institute of Anatomy and Cell Biology -Salzburg, Paracelsus Medical University, Salzburg, Austria; b College of Optometry, State University of New York SUNY, New York, USA

**Keywords:** Choroid-sclera transition zone, Myopia, Chicken, Marmoset, Human, TEM

## Abstract

Emmetropization is an active and visually guided process that involves the retina, choroid and sclera, and results in compensatory changes in eye growth. This guided growth is the result of visual cues and possibly mechanical interactions being translated into growth signals via molecular events from the retina into the choroid and sclera, through the choroidal scleral transition zone. If mechanical interactions were a part of the choroid-sclera signaling transduction cascade, specific morphological arrangements should be detectable in this region at the ultrastructural level. The goal of this study was to investigate the ultrastructural features of the choroidal scleral transition zone by comparing avian, non-human primate and human eyes, with the goal to confirm whether specific mechanical structures are present. Choroidal and scleral tissue from chicken, marmoset, and human eyes were imaged using transmission electron microscopy to document the choroid-sclera transition zone. In chicken eyes, fibroblast lamellae bordered the scleral matrix and formed thin end elongated processes that were undercut by scleral collagen fibrils. These processes back-looped into the scleral matrix, and displayed small club-like membrane protrusions. Differences in these arrangements in mature vs young chickens were not detected. The club-like membrane protrusions identified in chickens were rare in marmoset eyes, which instead exhibited two types of collagen fibrils discriminated by size, and were absent in the human eyes investigated. In marmoset and human eyes, elastic components were detected in the transition zone that were absent in chickens. In summary, cellular/membrane specializations indicating a mechanical interaction at the choroid-sclera transition zone were not detected in chicken, non-human primate or human eyes. If mechanotransduction is necessary for scleral growth, matrix integrity or development, alternative structural arrangements might be required.

## 1 Introduction

Emmetropization is an active process in charge of ensuring an adequate match between the biometric and optical features of the eye that can lead to myopia (nearsightedness) or hyperopia (farsightedness) when the image fails to fall on the retina and focuses in front or behind the retina, respectively [1]. Myopia is a complex and multifactorial condition [2], and is associated with an increase in ocular complications due to the increased eye size of myopic eyes [3]. Mild forms of myopia can be treated with contact lenses or glasses, resulting in a high socio-economic burden [4], but larger degrees of myopia, which affects approx. 10% of all myopes [5] are associated with sight-threatening ocular diseases such as glaucoma [6], cataract [7] and retinal detachment [8]. According to the World Health Organization, it is anticipated that by 2050 more than 50% of the world population will be myopic [9].

Experimental models of myopia have confirmed that visual input is responsible for post-natal eye growth and emmetropization [2, 10], and have begun to identify key genetic and molecular elements involved in the myopia signaling cascade [11]. This signaling cascade includes the retina, choroid and sclera, the last of which represents the end ocular growth target [12]. Imposing varying degrees of defocus on species ranging from rodents to non-human primates (NHP) [13] results in compensatory changes in eye growth and refractive state that act locally [14] and independently from the eye-brain axis [15]. Work on experimental models have also confirmed that the choroid represents an important relay tissue in this signaling process [16], further evidenced from a variety of pharmacological experiments [17, 18, 19, 20]. While major genes and canonical pathways have been identified in the retina and choroid [11], the mechanisms that translate visual signals into scleral growth remain unknown. Biochemical signals are believed to be involved in the first part of this transduction process across the retina, retinal pigment epithelium and choroid, due to the fast nature of the response [21, 22]. However, the signals involved in the delayed scleral response remain unexplored. We hypothesize that the scleral response is associated with mechanosensitive pathways. The choroidal scleral interface is a region of interest, as it represents where both mechanical and biochemical signals are expected to converge and influence final scleral growth [23]. Mechanical forces can be transduced by specialized organs connected to the autonomic nervous system, or via specialized structures within the cell surface (e.g., desmosomes, hemi-desmosomes, gap-junctions, or certain intracellular components [24, 25, 26]). Whether the choroidal scleral interface exhibits specialized structures that respond to visual signals and translates them into eye growth remains unexplored. The aim of this study was to analyze the choroidal scleral interface at the ultrastructural level using transmission electron microscopy in two well-established species of experimental myopia research, the chicken and the marmoset (Callithrix jacchus) [13], and compare to humans, to identify particular surface structures or membrane specializations that could serve as mechanosensitive trigger structures in the choroidal scleral interface.

## 2 Methods

**Figure 1:**
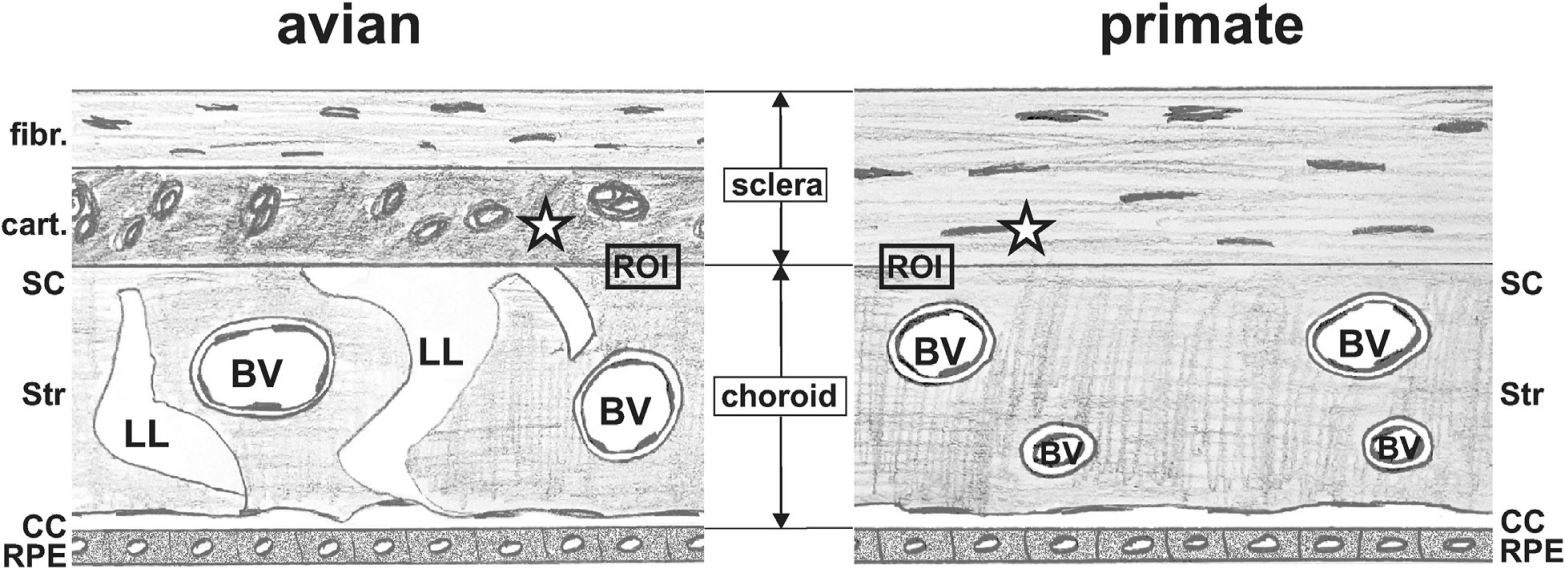
Overview of the region of interest investigated. In this sketch, an overview of the region of interest (ROI) investigated is given. The asterisks in the sketch reflect the asterisks in the micrographs and represent the site of scleral matrix in the transition zone. Note the difference in scleral composition in avians vs. primates: in avians, an inner cartilaginous (cart.) layer is formed by chondrocytes, while the outer fibrous (fibr.) layer is formed by fibroblasts. In contrast, the primate sclera is formed by fibroblasts only. BV: blood vessels; LL: lymphatic lacunae; SC: suprachoroid; Str: choroidal stroma; CC: choriocapillaris; RPE: retinal pigment epithelium.

Eyes from White Leghorn chicken (Gallus gallus, 14 days and 14 weeks old; n = 2), one common marmoset (Callithrix jacchus, male, 7 months old) and two human donors (female 90yrs, pm-time 23hrs; male 65yrs, pm-time 20hrs) were investigated. Human tissue was received from the body donation program, Institute of Anatomy and Cell Biology, Paracelsus Medical University, Salzburg, Austria, in full accordance with the Helsinki protocol and approved by the Salzburg State Ethics Committee (415-EP/73/775–2018 and EK1012/2019). Animal eyes were obtained in full accordance with the ARVO-regulations for the use of animals in ophthalmic and vision research and were obtained from collaborators. Eye cups were prepared for ultrastructural analysis by sectioning at the ora serrata and removing the vitreous body and retina with care to avoid lifting the underlying choroid. These eye cups (with the choroid attached to the sclera) were fixed in phosphate buffered saline (PBS) containing 4% paraformaldehyde and 2% glutaraldehyde (Sigma-Aldrich, Vienna, Austria) for 48 hrs at room-temperature. Tissue samples of size 5 × 5 mm were dissected and post-fixed in 1 % osmium tetroxide. Following dehydration in graded alcohols series, samples were embedded in Epon and mounted on Eponblocks. Semithin sections were stained with methylene blue, and silver-grey ultrathin sections were contrasted with lead citrate and examined in a transmission electron microscope (Leo112, Zeiss, Oberkochen, Germany) with a digital camera attached (2K wide angle slow scan CCD camera, TRS, Moorenweis, Germany) using the ImageSP software (SysProg, Minsk, Belarus). Whenever necessary, micrographs were adjusted in brightness and contrast for better visualization and documentation (Corel Draw 2018, Corel Corporation, Ottawa, Canada). A magnification index of all figures provided is given in Table 1. In order to facilitate orientation, an overview of the region of interest investigated is given in Figure 1.

**Table 1:** Magnification index of figures provided.

figure number	species	magnification
1A	Gallus gallus	Semithin section (160x)
1B	Gallus gallus	12500x
1C	Gallus gallus	6300x
1D	Gallus gallus	16000x
2A	Gallus gallus	12500x
2B	Gallus gallus	12500x
2C	Gallus gallus	25000x
2D	Gallus gallus	25000x
2E	Gallus gallus	12500x
3A	Callithrix jacchus	10000x
3B	Callithrix jacchus	12500x
3C	Callithrix jacchus	12500x
3C inset	Callithrix jacchus	80000x
4A	human	6300x
4B	human	3150x
4C	human	12500x

## 3 Results

**Figure 2:**
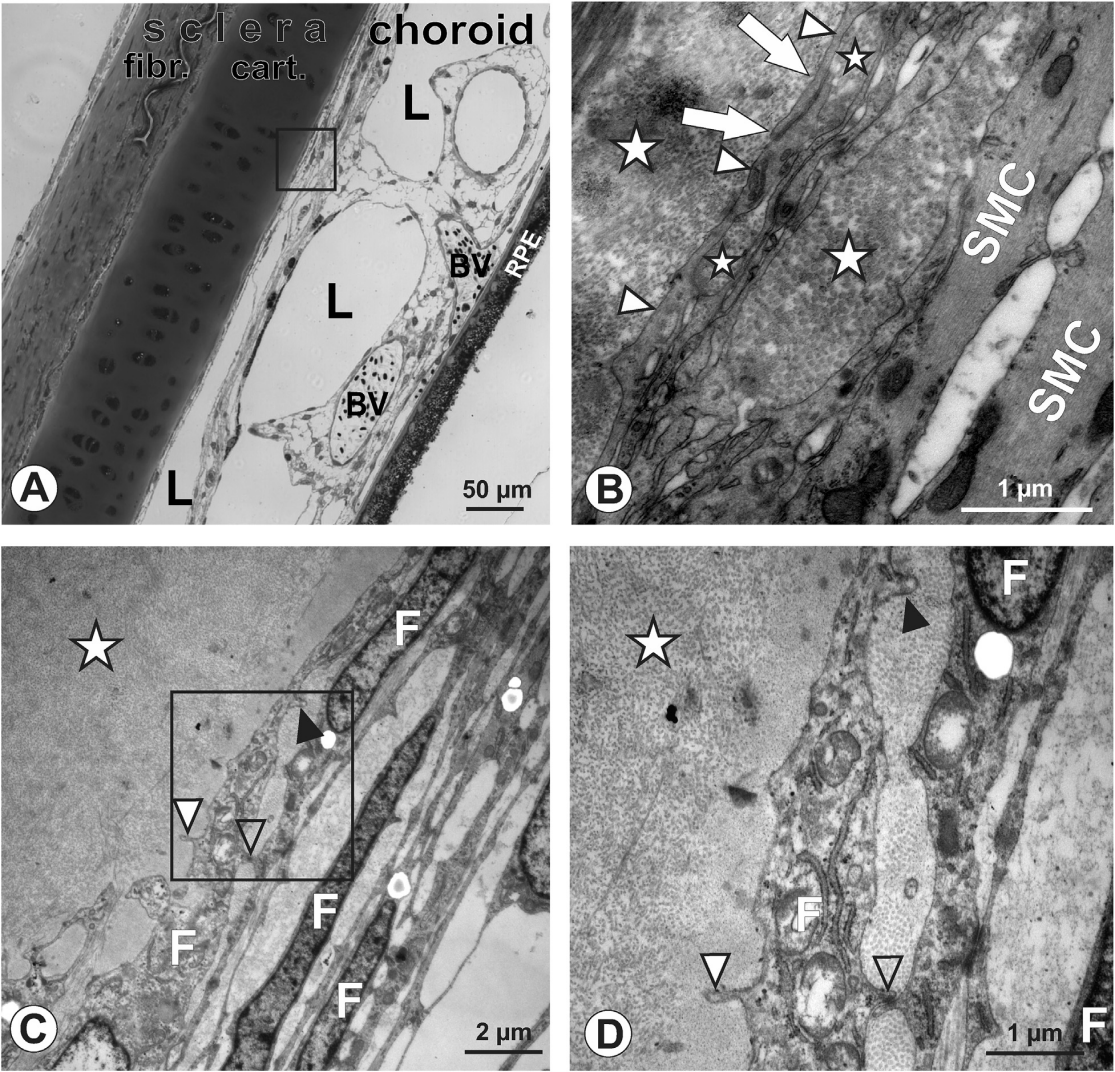
Chicken, 14 weeks old. A: Semithin section of the chicken sclera-choroid complex, overview: The sclera with its inner cartilaginous part and outer fibrous part and the adjacent choroid is clearly discernible, with the choroid-sclera transition zone intact. L depicts lymphatic lacunae, BV: blood vessels (containing avian nucleated erythrocytes), RPE: retinal pigment epithelium. Boxed area marks the localization of the further investigated sclera-choroid transition zone (valid for all species). B: Thin and long processes of fibroblasts form the sclera-choroid transition zone (arrowheads), connected with membrane specializations representing tight junctions (arrows). Scleral collagen fibrils (asterisks) undercut the fibroblast lamellae, reaching also into deeper layers that contain non-vascular smooth muscle cells (SMC; as identified by their intracellular striated pattern of actin-filaments). C, D: When closely investigating the fibroblast front-line lamella facing the sclera (asterisk in C, D), fibroblast (F) club-like membrane protrusions into the scleral matrix were detected (white arrowheads in C, D) as well as in the undercutting scleral collagen fibrils (black arrowheads in C, D), that were reminiscent of hook-and-loop like fasteners (with protrusions representing the hook, and the collagen matrix the loop). Fibroblasts itself were attached via tight junctions (open arrowhead). Micrograph D represents a magnification of boxed area in C. A putative difference in size of collagen fibres in chicken as seen in 1 B- to D has been interpreted as optical effect due to fibre orientation.

**Figure 3:**
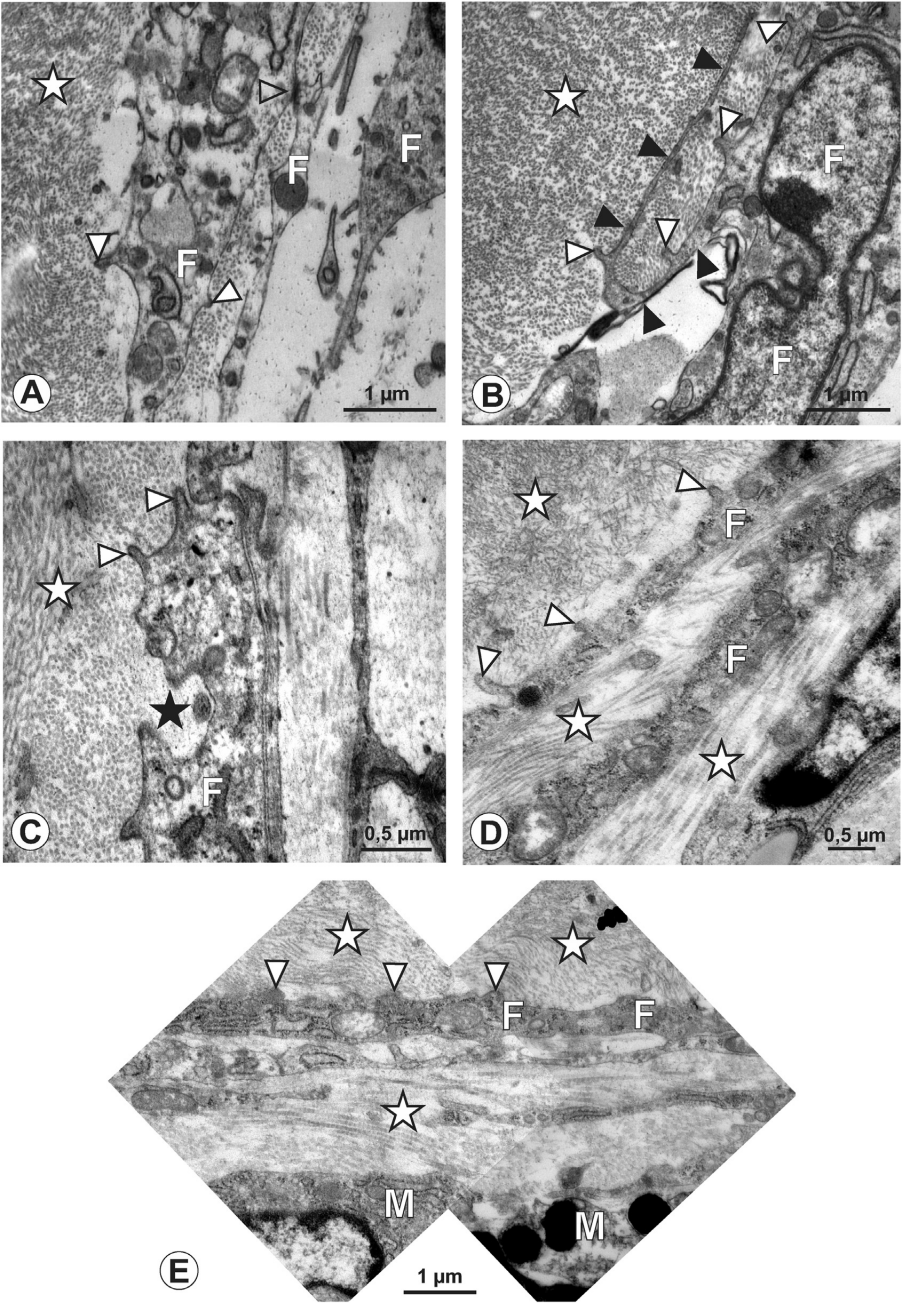
Chicken, 14 weeks old. A: Fibroblast processes (F) facing the scleral collagen matrix (asterisk) develop club-like membrane protrusions, their arrangement reminiscent on a hook-and-loop-like fastener. These protrusions invade the sclera, at both their outer (facing sclera) and inner (facing choroid) side (arrowheads). Fibroblasts are connected via tight junctions (open arrowhead). B: Fibroblast (F) forming the border lamella to the scleral matrix (asterisk) develop sprout-like looping process (black arrowheads) with club-like membrane protrusions (white arrowheads) on the surface of the cell body as well as its process. C: Occasionally, the bordering fibroblast lamella (F) facing the sclera collagen matrix (white asterisk) broadens and forms intrusions (black asterisk) filled with scleral matrix particles. Again, club-like membrane protrusions are detected on the scleral side (white arrowheads). Chicken, two-week old. D, E: In two-week-old chickens, a similar situation was detected as in mature ones: fibroblast lamellae (F) bordering the scleral matrix (asterisks) formed club-like membrane protrusions (arrowheads in D), sometimes also forming “less developed” protrusions with shorter loop and broader base (arrowheads in E). Note the decreased scleral matrix density in young animals as compared to mature animals. M: melanocyte.

Chick eyes, regardless of age, exhibited a bipartite sclera with outer (fibrous) and inner (cartilaginous) portions (Figure 2A, semithin section). In the old chick eye (14 weeks), the choroid was attached to the adjacent retinal pigment epithelium and the choroid-sclera transition zone intact. In the ultrathin sections, this transition zone consisted of three to five layers of thin and elongated processes of fibroblasts that formed a clear border with the scleral matrix, as identified by its collagen fibrils of uniform diameter (Figure 2B). Oriented towards the choroid, these fibroblasts were attached to non-vascular smooth muscle cells, which were identified based on its striated cytoplasm representing actin-filaments (Figure 2B). Layers of fibroblast processes were undercut by collagen fibrils originating from the scleral matrix, forming the transition zone proper (Figure 2B-D). Fibroblast processes in direct contact with the scleral matrix (i.e. those ones forming the bordering lamella) exhibited a smooth and uniform cell border without further specializations, except on those portions were there was contact with another fibroblast, where tight junctions were present (Figure 2D). On certain areas of the bordering lamella, small club-like membrane protrusions (approx. 0.25 μm in length) reached into the scleral matrix (Figure 2C, D, Figure 3A). These club-like membrane protrusions were also present on the fibroblast side and reached into the undercutting scleral matrix, which was oriented towards the choroid (Figures 2D, 3A). We also detected bordering fibroblasts that developed long and sprout-like processes looping into the scleral matrix with additional club-like membrane protrusions (Figure 3B). In several sections, we observed bordering fibroblasts that developed broader processes forming intrusions filled with scleral matrix (Figure 3C).

The younger chick eye (14 days) exhibited structures that were similar to the ones identified in the older chick (Figure 3D, E): bordering fibroblasts developed club-like membrane protrusions of similar size, appearance and frequency as in the mature chick eye. In addition, processes of bordering fibroblasts were also undercut by collagen fibrils that originated from the scleral matrix (Figure 3D). We did, however, observe some age-related differences: the scleral matrix in the younger 2-week-old chicken was looser, more disorganized and less condensed compared to 14-week-old chicken (Figure 3D, E). Further, certain sections of the bordering fibroblast processes were also different in the 2-week-old chickens: the hook-and-loop protrusions appeared less elongated and broadened at their basal side (Figure 3E).

**Figure 4:**
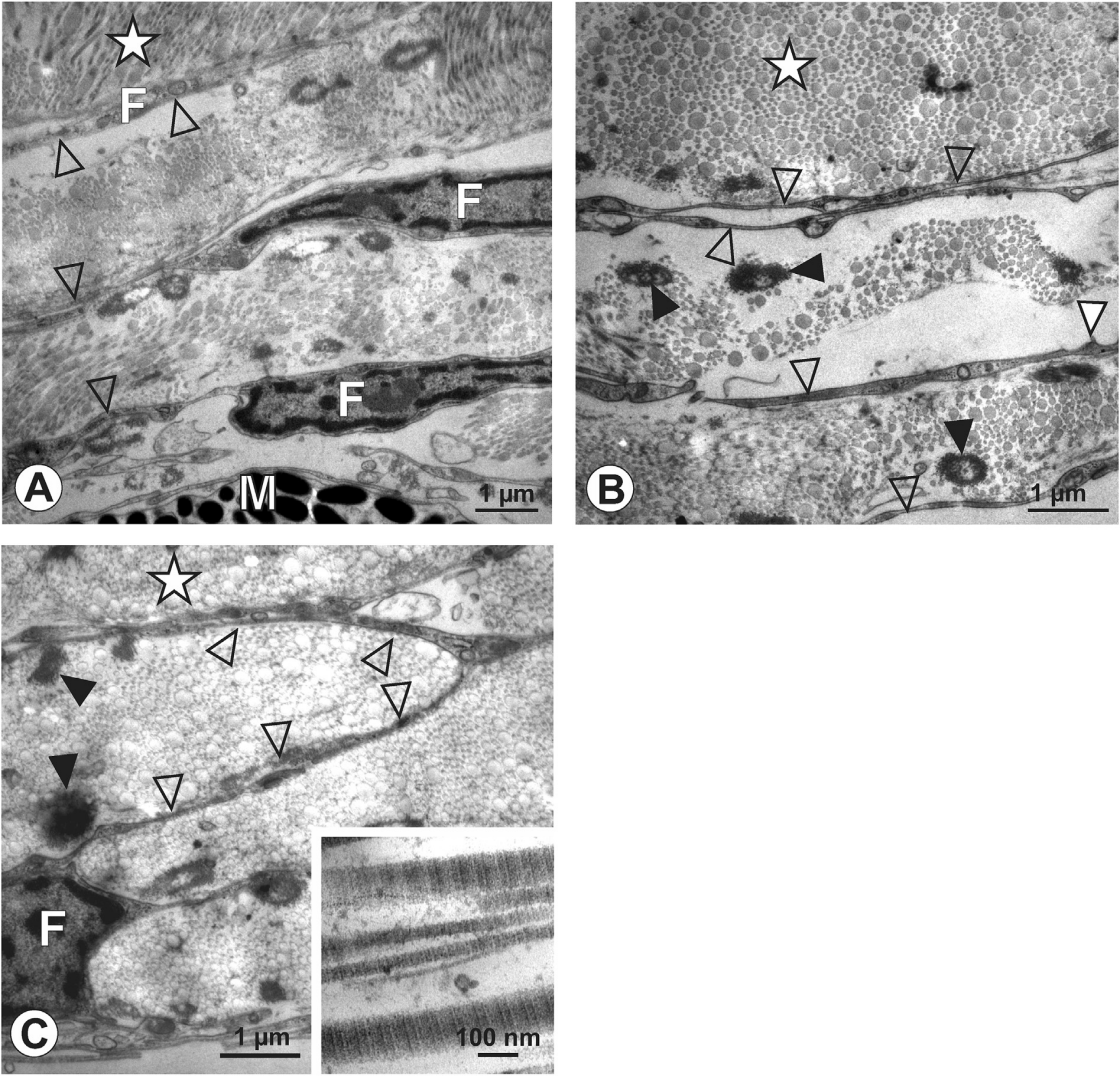
Marmoset, 7 months. A to C: In marmosets, fibroblast (F) lamellae (open arrowheads) bordering the scleral matrix (asterisk) were extremely slender, immediately decreasing their diameter when leaving the nuclear region of the cell (open arrowheads in A, B). Club-like membrane protrusions were only occasionally detected (white arrowhead, B). Scleral collagen matrix undercut the fibroblast processes and consisted of large and small-type diameter collagen fibrils (B, C, and inset in C). Occasionally, electrondense material was found enclosed in the collagen matrix, and represents portions of elastic fibers (black arrowheads, B, C). Sprout-like looping processes emerged from fibroblasts into the scleral collagen matrix (open arrowheads, C). M: melanocyte.

In the marmoset (Figure 4A-C), the scleral-choroid transition zone showed a similar arrangement as in chickens: three to five fibroblasts processes formed several lamellae bordering the scleral matrix (Figure 4A), and collagen fibrils of the scleral matrix undercut these processes (Figure 4A, B). The arrangement of the fibroblast processes, however, was different: compared to the chicken eye, in the marmoset these were extremely slender and almost immediately thinned to their final diameter when leaving the nuclear region of the cell. Club-like membrane protrusions were observed but were less frequent than in the chick (Figure 4B). The collagen content of the matrix differed from chickens: two types of collagen fibrils were discernible in cross sections as well as in longitudinal sections (Figure 4B, C, inset in C). The overall diameter was approximately 25 ​nm, some of them reaching 100 ​nm in diameter. Processes with sprout-like endings that looped into the scleral matrix were observed in marmosets as well as in chick eyes (Figure 4C).

**Figure 5:**
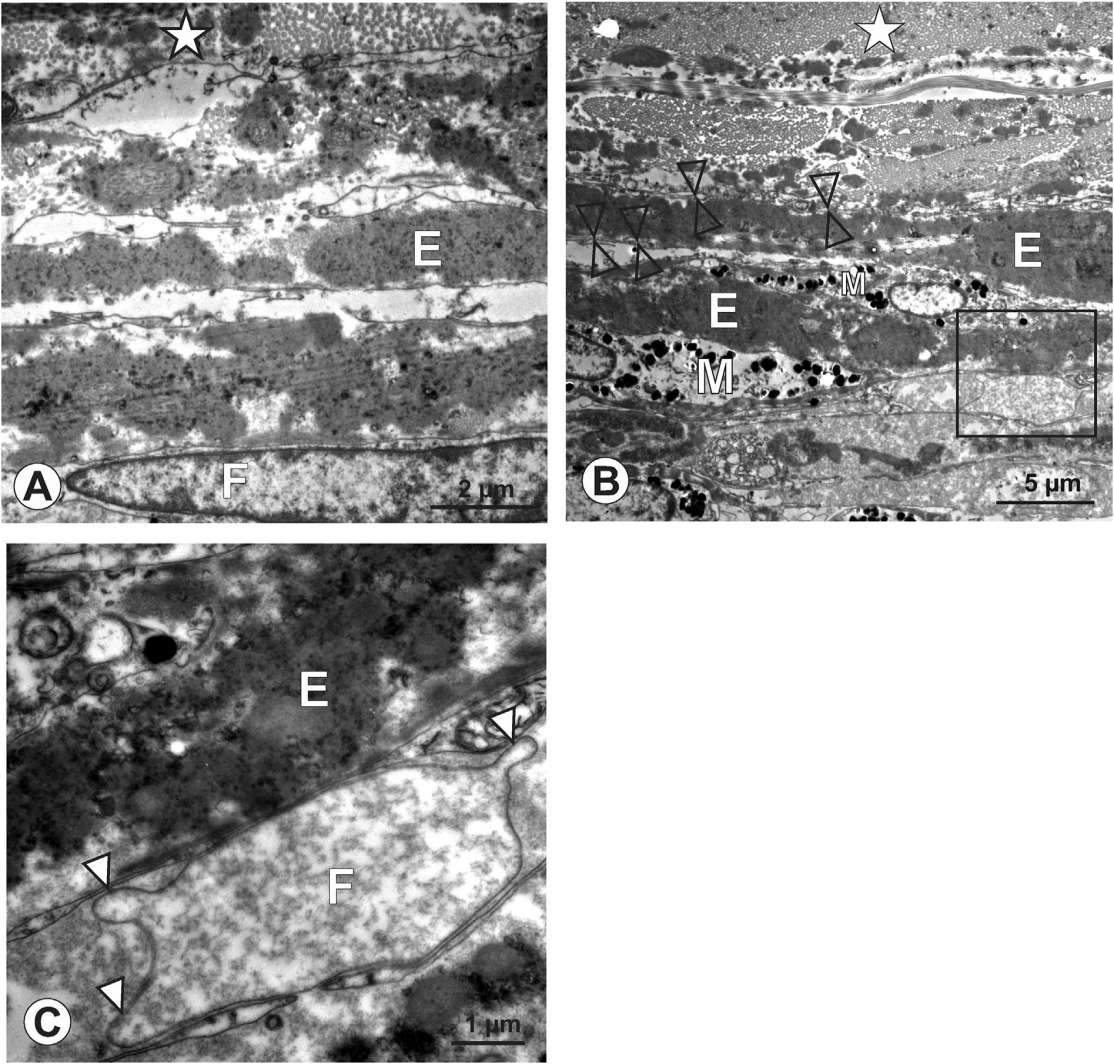
Human, 90 years. A to C: In this female individuum, bordering fibroblast (F) lamellae separated the collagen matrix of the sclera (asterisk in A, B) and were slender and elongated (A, B open arrowheads). The collagen fibers were of uniform diameter (A, B), and an elastin-rich layer was embedded in the transition zone (E in A to C). Club-like membrane protrusions were only occasionally detected and if present, displayed more circumferentially orientation within the scleral matrix (C, arrowheads). Micrograph in C represents a magnification of the boxed area in B. M: melanocyte.

In human eyes (Figure 5A-C), the transition zone adjacent to the scleral collagen fibers displayed a different arrangement than in the other two species investigated: bundles of elastic fibers were present, building an elastic layer cluster of roughly 10 μm in diameter (Figure 5A, B), that gradually decreased towards the scleral side. Extremely slender fibroblast processes were detected between these elastic components (Figure 5B), together with processes deriving from melanocytes, as identified by their content of electron-dense melanin granules. Scleral collagen fibers displayed a uniform diameter (Figure 5B). Unlike in chick and marmoset eyes, in human eyes we did not observe club-like membrane protrusions when trailing the fibroblast processes bordering the scleral collagen fibrils. Occasionally, such protrusions were detected in deeper layers of the transition zone (Figure 5C), however, these were more circumferentially oriented instead of running exclusively towards the scleral collagen fibers.

## 4 Discussion

This cross-species study investigated the choroid-sclera transition zone at the ultrastructural level with the goal to identify cellular structures that might be involved in the emmetropization pathway that eventually leads to changes in scleral biomechanics and scleral growth. We characterized two well-established experimental models of myopia and compared them with human eyes and provide additional information on the choroid-scleral transition zone that can be considered important for scleral biomechanics [27].

In anatomy textbooks, the sclera is described as a three layer tissue comprised by an external episclera, an intermediate stroma responsible for the sclera's biomechanical properties [28], and an innermost lamina fusca adjacent to the suprachoroid. Melanocytes accumulate in this layer for yet unknown reasons. Their melanin granules are dark-brownish (latin “fuscus”) and help discriminate from the melanocyte–free remaining parts of the sclera [27]. Interestingly, some authors consider the lamina fusca a part of the suprachoroid [16, 29], thus highlighting that the boundary between the sclera and choroid remains unclear. While a distinction can be made using the fibroblasts present at the choroidal scleral transition zone, a more precise approach is to use the electron microscope to identify architectural landmarks: a matrix with strictly organized collagen lamellae is the sclera hallmark, whereas a less organized matrix with intermingling collagen fibrils and rich in ground substance and loose connective tissue, is the suprachoroid. The location of the matrix with regards to the transition zone allows for a better discrimination between the sclera and choroid, irrespective of the fibroblast origin (scleral vs. choroidal). Hence, the bordering cells within this matrix have been considered the transition zone proper. This is valid for many vertebrate species, including non-human primates like marmosets and humans, and it also applies to species with an inner scleral matrix formed by chondrocytes, as it is the case in chickens [30].

While the choroid and sclera have been studied at the ultrastructural level in many species [29, 31, 32, 33, 34], the choroid-sclera-transition zone, to the best of our knowledge, has not been investigated. Furthermore, data describing the posterior sclera/uvea in marmosets does not exist. In all three species investigated in this study, the bordering structures from the choroid to the sclera were stacks of fibroblasts [16]. These formed thin and elongated lamellae that reached into the scleral collagen matrix and were undercut by scleral collagen fibrils, which formed an additional bonding that might serve to resist intraocular mechanical forces. This arrangement was enhanced by the observed back-looping fibroblast branches seen in chicken and marmosets. However, this arrangement was not detected in humans, which might be counterbalanced by the presence of elastic components in the human transition zone. Elastic components are found in the sclera-choroid interface of many species [35, 36, 37, 38, 39]. In this study, they were most prominent in the human eyes, present but less pronounced in the marmoset, and absent in chickens. This might be interpreted as a lack of need to translate elastic forces in avian eyes, as shown by their cartilage layer. However, additional mechanical anchor points are necessary in the transition zone of the avian eye, and accomplished by the presence of the club-like membrane protrusions (filled arrowheads in Figure 2C, D). When the authors first observed them, they reminded of hook-and-loop-fasteners; protrusions representing the hook, and the collagen matrix the loop, possibly contributing to mechanisms responding to mechanical changes. These structures were infrequent in marmoset, and completely absent in humans.

Fibroblasts form most of the cellular structures identified in the transition zone of most species [28]. While melanocytes can be found within this transition zone, in this study they did not create the final bond with the scleral matrix. In fact, their function within transition zone remains unknown [40]. Their melanin content might serve to absorb back-scattered light from deeper scleral layers, thus avoiding interference with direct retinal signals. However, a more pronounced accumulation of these cells would be required. Similarly, they could represent a remnant of invaded cells during ocular development [41, 42]. Non-vascular smooth muscle cells can be observed in the avian choroid [29], are also present in the choroid of many other species including human [43, 44, 45, 46, 47], do not belong to the transition zone and are a part of deeper layers. Most likely, they contribute to the contraction and relaxation of the tissue, a mechanism that has been termed *choroidal accommodation* [10, 30, 48]. However, the control underlying this mechanism (e.g., visually guided or controlled by the autonomic nervous system, or both), remains unknown [16]. Other authors have suggested the presence of a unique cell population termed telocyte-like cells in the transition zone [49, 50]. While they might be considered mechanosensors or stem-cell niche in other organs [51], a deeper understanding of this cell type and its function is required, which was beyond the scope of this work.

With respect to the differences identified between the matrices of chick and marmoset, we speculate that they relate to anatomical differences in the primate vs avian eye. In the latter one, a stiffer sclera is present supported by hyaline cartilage and/or the presence of scleral ossicles [52]. This feature might be related to different mechanical forces imposed by corneal accommodation in the avian eye [53]. The elastic fibres identified in both marmoset and humans suggest that more elasticity and less stiffness might be necessary for an adequate ocular physiology and function. While it is commonly accepted that a decrease in elastic matrix properties takes place during aging [54], and this holds also true for the choroid [55], only minor changes in biomechanics have been described in aged human eyes [56]. Elastin has been detected in avian eyes during ciliary body development but not in the choroid [57], was not observed in this study, but has been described at the ultrastructural level in adult chick eyes [29]. Whether this difference is age-related, or associated with topographical differences in locations with particularly demanding mechanics, remains unexplored [58, 59].

Interesting were the differences in collagen caliber observed in the marmoset sclera. Type 1 collagen is the major collagen component in the sclera in many species including the avian fibrous sclera [60, 61]. The differences in caliber observed in marmosets might describe mechanistic differences to respond to various mechanical strain in this region, as shown by mechanical in-vitro experiments: larger diameter of collagen fibrils are associated with a higher resistance to deformation at low strain, while longer fibrils may be important for the ultimate mechanical properties at high strain [62].

While structural differences might exist with age, it was not the goal of this study to characterize age-related effects. Two-week-old chick eyes were selected because ocular growth rates peak at this age. We speculated that any morphological peculiarity at the scleral-choroid-interface, if present, would reach a maximum at this age and more obvious when compared to mature animals. This however, was not the case in chickens.

During the last decades, it has become clear that the choroid represents a crucial tissue during emmetropization to relay the retinal visual information to the sclera [63]. The signal cascades in these pathways are not yet fully understood, and we hypothesized that membrane specializations or cellular peculiarities would be present to provide mechanical transduction within the choroidal scleral transition zone. However, the only specialization we detected in this transition zone were membrane protrusions reminiscent on hook-and-loop-like connections. These were most prominent in chickens, occasionally found in marmosets, but absent in humans. We also hypothesized that if such specializations were present and meaningful, their numbers should be increased in the developing eye. Since no differences were detected between mature and young chicken eyes, and the amount of hook-and-loop-like connections were the same in the mature vs young chicken we propose that mechanical transduction is not managed via these structures. In line with our assumption is the absence of such structures in marmoset and human eyes. The marmoset was 7 months old, which is equivalent to childhood in humans. Both marmoset and human eyes continue to grow at this age to emmetropize. While eyes from human adolescent subjects were not available for obvious reasons, this is a drawback of this study, and future studies might be able to characterize the choroidal scleral interface in human growing eyes.

The scleral matrix differences described in this study might relate to the various ocular growth rates exhibited by the animals studied [2], which in turn might have an effect on the choroid (e.g., non-vascular smooth muscle cells [29, 46], lymphatic lacunae [64, 65], intrinsic innervation [66, 67, 68]) and the physiology of the choroid-sclera borderline region. However, these associations remain unexplored. In this respect, while the visual signal transduction most likely occurs via molecular or chemical pathways, a mechanical component should not be overlooked. At least for the developing eye and sclera, the theory of causal histogenesis for connective tissue structures [69] remains plausible; fibroblasts and chondrocytes act as receptors in the skeletal and also the visual system and sense, adapt and respond to mechanical forces with matrix formation. Therefore, pure force transduction without particular cell surface specialization would be possible. The mechanical anchor points identified in this study (hook-and-loop-like structures and looping branches in chickens) may influence scleral matrix formation, as would also the general mechanical force transduction in the absence of such structures (marmoset, human). In this sense, mechanical and biochemical signals from the outer suprasclera or even outside the eye might contribute to matrix adaption: transscleral delivery for some molecules is established [70] and mechanical forces could be delivered also via the origin/insertion-point of the extraocular muscles tendons [71]. Still, the critical factors and influencing systems of the various tissues involved in regular/irregular ocular development and their interaction(s) are not understood and await further clarification. Nevertheless, alterations of choroidal and scleral structure and biomechanics in myopia will be of great interest and might contribute to the understanding of changes in myopic eyes upon ageing.

## Declarations

### Author contribution statement

Platzl C, Kaser-Eichberger A, Benavente-Perez A, Schroedl F: Conceived and designed the experiments; Performed the experiments; Analyzed and interpreted the data; Contributed reagents, materials, analysis tools or data; Wrote the paper.

### Funding statement

This work was supported by 10.13039/501100004061Austrian National Bank Fund, ÖNB#17617, and the Research Fund of 10.13039/501100011852Paracelsus Medical University, R-16/04/085-SCK.

### Data availability statement

No data was used for the research described in the article.

### Declaration of interests statement

The authors declare no conflict of interest.

### Additional information

No additional information is available for this paper.
